# Factors associated with older persons’ perceptions of dignity and well-being over a three-year period. A retrospective national study in residential care facilities

**DOI:** 10.1186/s12877-022-03205-1

**Published:** 2022-06-23

**Authors:** Charlotte Roos, Moudud Alam, Anna Swall, Anne-Marie Boström, Lena Marmstål Hammar

**Affiliations:** 1 School of Health and Welfare, Dalarna University, Falun, Sweden; 2 School of Information and Engineering, Dalarna University, Falun, Sweden; 3 Division of Nursing, Department of Neurobiology, Care Sciences and Society, Karolinska Institute, Stockholm, Sweden; 4 Theme Inflammation and Ageing, Unit Nursing Ageing, Karolinska University Hospital, Huddinge, Sweden; 5 Research and Development Unit, Stockholms Sjukhem, Stockholm, Sweden; 6 School of Health, Care and Social Welfare, Mälardalen University, Västerås, Sweden

**Keywords:** Dignity, Long-term care, Older persons, Person-centred care, Person-centred practice framework, Residential care facilities, Well-being

## Abstract

**Background:**

Dignity and well-being are central concepts in the care of older people, 65 years and older, worldwide. The person-centred practice framework identifies dignity and well-being as person-centred outcomes. Older persons living in residential care facilities, residents, have described that they sometimes lack a sense of dignity and well-being, and there is a need to understand which modifiable factors to target to improve this. The aim of this study was to examine the associations between perceptions of dignity and well-being and the independent variables of the attitudes of staff, the indoor-outdoor-mealtime environments, and individual factors for residents over a three-year period.

**Methods:**

A national retrospective longitudinal mixed cohort study was conducted in all residential care facilities within 290 municipalities in Sweden. All residents aged 65 years and older in 2016, 2017 and 2018 were invited to responded to a survey; including questions regarding self-rated health and mobility, the attitudes of staff, the indoor-outdoor-mealtime environments, safety, and social activities. Data regarding age, sex and diagnosed dementia/prescribed medication for dementia were collected from two national databases. Descriptive statistics and ordinal logistic regression models were used to analyse the data.

**Results:**

A total of 13 763 (2016), 13 251 (2017) and 12 620 (2018) residents answered the survey. Most of them (69%) were women and the median age was 88 years. The odds for satisfaction with dignity did not differ over the three-year period, but the odds for satisfaction with well-being decreased over time. Residents who rated their health as good, who were not diagnosed with dementia/had no prescribed medication for dementia, who had not experienced disrespectful attitudes of staff and who found the indoor-outdoor-mealtime environments to be pleasant had higher odds of being satisfied with aspects of dignity and well-being over the three-year period.

**Conclusions:**

The person-centred practice framework, which targets the attitudes of staff and the care environment, can be used as a theoretical framework when designing improvement strategies to promote dignity and well-being. Registered nurses, due to their core competencies, focusing on person-centred care and quality improvement work, should be given an active role as facilitators in such improvement strategies.

## Background

Dignity is a central concept in the care of older people worldwide [1]. To preserve dignity, respect must be paid to a person’s integrity and self-determination, i.e., autonomy [2, 3]. As older people live longer with both comorbidities and long-term disabilities [4], it commonly implies restricted autonomy [5]. Individualized care is described as an important aspect to promote dignity [6]. Older persons living in residential care facilities (RCFs) have, in experiencing self-determination and individualized care, described the importance of having choices regarding one’s care. In addition, the importance of having control over how to receive care, when to receive care, [6–9] what to eat, when to eat and where and with whom to eat [10] have been emphasized. However, staff in RCFs sometimes lack the ability to promote self-determination and individualized care [7, 9–11]. Furthermore, well-being is a central concept in the care of older people worldwide, and International Sustainable Development Goal Number Three highlights the promotion of well-being for humans of all ages [12]. Well-being is described as a subjective feeling of pleasure [13]. To experience well-being, older persons living in RCFs (residents) have described the importance of meaningful activities, i.e., activities that agree with an individual’s hobbies and lifestyle. Nevertheless, residents have reported a lack of such activities in RCFs [14, 15].

In Sweden, dignity and well-being are central concepts in the legislation regulating the care of older persons receiving home care services and those living in RCFs. The legislation regarding dignity and well-being is named the Swedish national fundamental values (SNFVs) of older persons [16, 17]. The SNFVs define that in order for residents to experience dignity, staff members must respect residents’ personal integrity, self-determination and participation. To experience dignity, it is further important that care is individualized and that staff members treat residents with a respectful attitude. To experience well-being, according to the definition in the SNFVs, residents must feel meaningfulness and safety. The Swedish National Board of Health and Welfare (NBHW) was assigned to facilitate the implementation of the SNFVs in all RCFs in Sweden when it was legislated [18]. One strategy used was the development of educational material to be used by staff members in RCFs [19]. Another strategy was that managers of the RCFs were invited to participate in a university course regarding how to support and facilitate the implementation of the SNFVs [20]. Every year, the Swedish NBHW conducts a survey of all residents aged 65 years and older. The survey aims to capture residents’ perceptions regarding their care [21]. Despite the strategies used to implement the SNFVs, the results from the survey between 2012 and 2015 indicated that far from all residents were fully satisfied with experiencing aspects of dignity and well-being [22, 23]. However, the SNFVs were legislated in 2011 [16], and it is well known that implementing new knowledge is difficult [24].

**Fig. 1:**
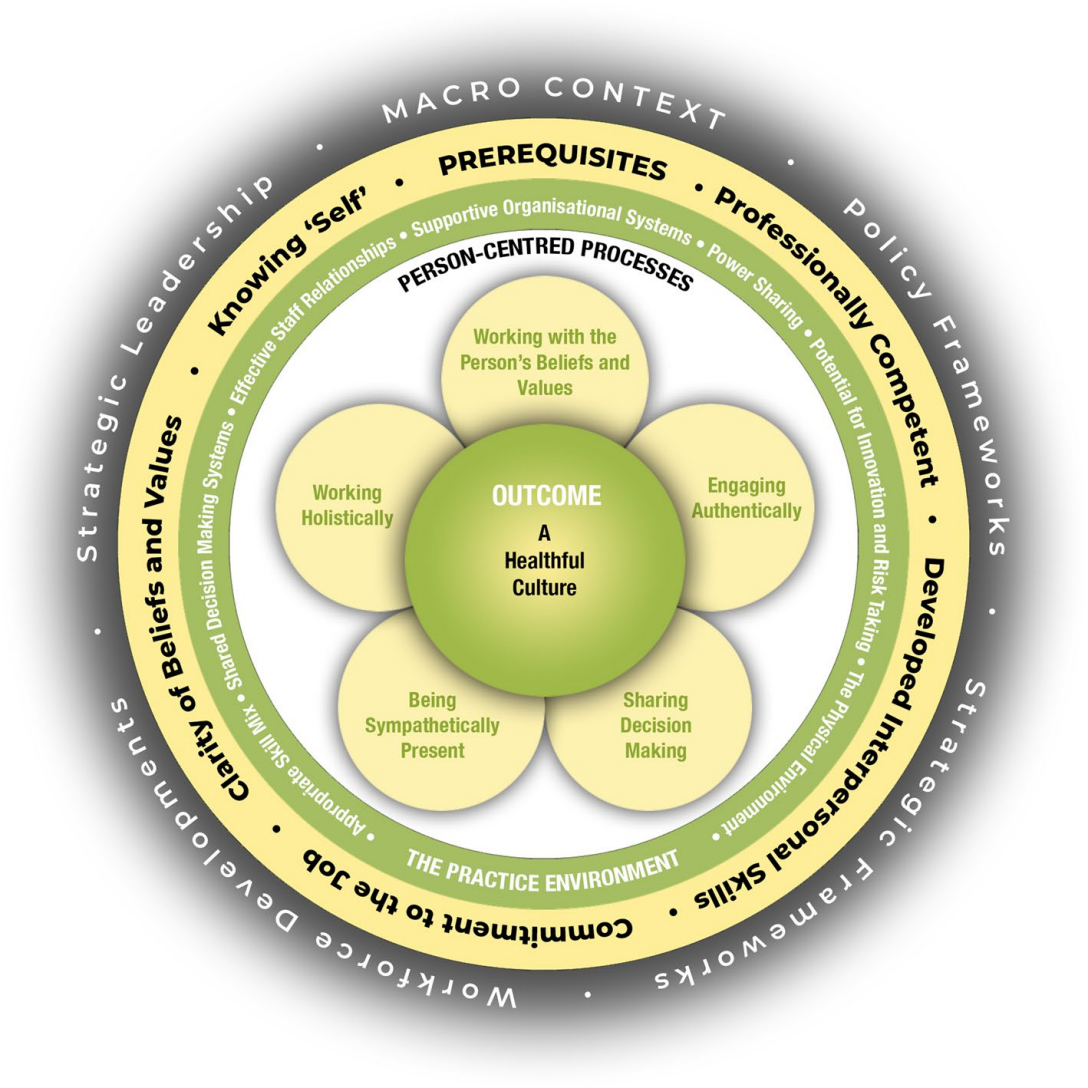
The Person-centred practice framework

Dignity and well-being can be interpreted as person-centred outcomes according to the person-centred practice (PCP) framework [25, 26]. This means that the PCP framework could be used as a theoretical framework when implementing the SNFVs of dignity and well-being in RCFs. The PCP framework contains the constructs of the *prerequisites of staff* (knowledge, skills, and attitudes), *care environment* (the context where care is provided), *person-centred processes* (delivering care by having a clear picture of the person’s beliefs and what the person values in his/her life) and *person-centred outcomes.* The prerequisites are influenced by the care environment, and both of these in turn influence the person-centred processes leading to the person-centred outcomes of dignity and well-being [25, 26], see Fig. 1 for an overview of the PCP-framework.

The researchers of the present study have recently, departing from the PCP framework, conducted a cross-sectional study in RCFs. In the cross-sectional study it was found that to promote dignity and well-being, the following factors need to be targeted: the attitudes of staff, the indoor-outdoor-mealtime environments, self-rated health, mobility and diagnosed dementia/prescribed medication for dementia. These factors were associated with perceptions of dignity and well-being [27]. The recent cross-sectional study was conducted in 2018 and provided guidance regarding what factors to target to promote dignity and well-being. In addition, there is a need to know if these factors are persistent over time. This is important to know in order to designing sustainable improvement strategies to promote dignity and well-being. Departing from the PCP framework, the aim of this study was to examine the associations between perceptions of dignity and well-being (dependent variables) and the attitudes of staff and the indoor-outdoor-mealtime environments (independent variables) over a three-year period. Individual factors for residents, such as self-rated health, mobility and diagnosed dementia, are not considered in the PCP framework, but in previous research conducted in both home-care settings and RCFs, it was found that persons perceiving poor health and persons diagnosed with dementia had higher odds of being more dissatisfied with aspects of dignity [27, 28]. As most of the residents living in RCFs in Sweden have an extensive need for care due to poor health and diagnosed dementia [29, 30], we need to increase our understanding regarding whether these factors are associated with perceptions of dignity and well-being over time. Thus, the factors of self-rated health and dementia were also taken into consideration in this study. Our hypothesis was that residents’ perceptions of satisfaction with dignity and well-being over a three-year period are associated with a) the attitudes of staff, b) the indoor-outdoor-mealtime environments and c) individual factors. Perceptions of a) respectful attitudes of staff, b) thriving in the indoor-outdoor-mealtime environments and c) good health are associated with higher satisfaction regarding dignity and well-being over a three-year period.

## Methods

### Study design

A retrospective longitudinal mixed cohort study design [31] using self-reported data from the national survey by the NBHW in 2016, 2017 and 2018.

### Participants and setting

The study was performed in RCFs in Sweden. In Sweden the municipalities are responsible for providing RCFs to older persons and the RCFs are funded by taxes [16]. RCFs provide one-room apartments, and there are also public indoor areas such as dining areas and outdoor areas [32]. In RCFs, care is provided around the clock by nurse assistants (NAs) and registered nurses (RNs) [33]. The older person living in a RCF pays rent for the apartment and a fee for care and services [32].

In the study, all residents aged 65 years and older living in all RCFs in the 290 municipalities in Sweden were invited to respond to a survey in 2016, 2017 and 2018. If a resident was unable to respond, a relative, friend, trustee, or staff member (proxy) was asked to respond instead. Answers from proxies were excluded from this study. An overview of the target population, self-respondents, and response rates is presented in Table 1.

**Table 1:** Overview of the target population, survey responses, self-respondents and response rates

Respondents	2016	2017	2018
Target population (n)	72,724	71,577	71,696
Survey responses: Self-respondents and proxies (n), (response rate, %)	40,371 (56%)	38,491 (54%)	35,432 (49%)
Self-respondents (n), (response rate, %)	13,763 (19%)	13,251 (19%)	12,620 (18%)

### Data collection

Data were collected using the national survey by the NBHW conducted in 2016, 2017 and 2018. The survey questionnaires were distributed by the NBHW. It was possible to answer the survey in a paper format or online. The survey contained 27 questions addressing the following areas: self-rated health, the indoor-outdoor-mealtime environments, the performance of care, the attitudes of staff, safety, social activities, the availability of staff and care in its entirety. The survey results are intended to be used for quality improvements in care [21]. When the survey was developed in 2012; a reference group provided input regarding the questions, the questions were tested by cognitive interviews, and researchers peer-reviewed the survey [23].

### Measures – dependent variables

Three survey questions with a focus on *personal integrity, self-determination, participation,* and *individualized care* were identified to measure the dependent variable of *dignity*. The identification of the questions was performed according to the definition of the SNFVs: for residents to perceive dignity, respect must be paid to their personal integrity, self-determination and participation, and care must also be perceived as individualized [18]. Two survey questions with a focus on *safety* and one question regarding *meaningfulness* were identified to measure the dependent variable of *well*-*being*. The identification of the questions was performed according to the definition of the SNFVs: for residents to perceive well-being, it requires that they experience both safety and meaningfulness. Table 2 provides an overview of the dependent variables and how the survey questions were linked to the SNFVs of dignity and well-being.

**Table 2:** Overview of the dependent variables and how the survey questions are linked to the SNFV of dignity and well-being

	**Model 1 Dignity**	**Model 2 Dignity**	**Model 3 Dignity**	**Model 4 Well-being**	**Model 5 Well-being**	**Model 6 Well-being**
Link to the SNFV of dignity and well-being	Respect for personal integrity. Participation. Individualized care	Respect for personal integrity. Self-determination. Participation. Individualized care	Respect for personal integrity. Self-determination. Participation. Individualized care	Safety	Safety	Meaningfulness
Question	Do staff inform you beforehand about changes in your care?	Can you influence what time to get care?	Do staff consider your opinions and wishes regarding your care?	How safe or unsafe does it feel to live in the RCF?	Do you feel trust in staff at the RCF?	How satisfied or dissatisfied are you with the social activities offered at the RCF?
Response alternative	Ordinal response treated as categorical variable 1 = Always 2 = Most of the time 3 = Sometimes 4 = Seldom 5 = Never	Ordinal response treated as categorical variable 1 = Always 5 = Never	Ordinal response treated as categorical variable 1 = Always 5 = Never	Ordinal response treated as categorical variable 1 = Very safe 2 = Quite safe 3 = Neither safe nor unsafe 4 = Quite unsafe 5 = Very unsafe	Ordinal response treated as categorical variable 1 = Yes, for all staff 2 = Yes, for most of the staff 3 = Yes, for some of the staff 4 = No, for none of the staff	Ordinal response treated as categorical variable 1 = Very satisfied 2 = Quite satisfied 3 = Neither satisfied nor dissatisfied 4 = Quite dissatisfied 5 = Very dissatisfied
Source	NBHW survey	NBHW survey	NBHW survey	NBHW survey	NBHW survey	NBHW survey

### Measures – independent variables

The independent variable *of time* was measured using data from the survey in 2016, 2017 and 2018. Two survey questions regarding health and mobility were used to measure the independent variable of s*elf-rated health*. To identify respondents with *dementia*, survey data were supplemented by two other national databases (the patient register and the medical register), also maintained by the NBHW. Respondents diagnosed with *dementia* were included in the patient register and identified with the ICD-10 codes F00-F003. Respondents with prescribed medication for dementia were included in the medical register and identified with the code N06D. Residents diagnosed with dementia and residents with prescribed medication for dementia will henceforth be named residents with dementia. In addition, data on *age* and *sex* were retrieved from the patient register. One survey question was used to measure the independent variable *of attitudes of staff*, where the respondents were asked to state if they had experienced any of ten negative incidents in their contact with staff. Four survey questions regarding the indoor (apartment and public indoor areas)-outdoor-mealtime environments were used to measure the independent variable of *care environment*. See Table 3 for an overview of the independent variables.

**Table 3:** Overview of the independent variables

Descriptions and Questions	Response alternatives and Measurement scales	Recoding	Source
***Individual factors***			
Age	1 = 65–79 years 2 = 80 years and older		Patient register
Sex	1 = Male 2 = Female		Patient register
Dementia diagnosis/prescribed medication for dementia	1 = Not Dementia 2 = Dementia		Patient register Medical register
How do you rate your health?	Ordinal response treated as categorical variable 1 = Very good 2 = Quite good 3 = Fairly 4 = Quite poor 5 = Very poor	1 = Good (1,2,3) 2 = Poor (4,5)	NBHW survey
How do you rate your mobility indoors?	Ordinal response treated as categorical variable 1 = I can move around by myself without difficulties 2 = I have some difficulties moving around by myself 3 = I have major difficulties moving around by myself 4 = I cannot move around by myself	1 = Can move around by myself (1) 2 = Difficulties/cannot move around by myself (2,3,4)	NBHW survey
***Attitudes of staff***			
Have you experienced any of the following in your contact with staff? 1.Did not show respect for your privacy, e.g., did not knock on the door before entering your room 2.Made negative comments about you, your belongings, or your home 3.Treated you disrespectfully in words or gestures 4.Treated you like a child 5.Denied your wishes for the help to be received 6.Denied your wishes at mealtimes 7.Did not show respect in toileting, bathing and dressing 8.Was harsh about toileting, bathing and dressing 9.Kept distance in nursing 10.Acted inappropriately in any other way	1 = Not experienced 2 = Experienced		NBHW survey
***Care environment***			
Do you thrive in your apartment?	Ordinal response treated as categorical variable 1 = Yes 2 = Partly 3 = No		NBHW survey
Are the public indoor areas pleasant?	Ordinal response treated as categorical variable 1 = Yes……3 = No		NBHW survey
Are the outside areas pleasant?	Ordinal response treated as categorical variable 1 = Yes……3 = No		NBHW survey
Do you experience mealtimes as a pleasant time of the day?	Ordinal response treated as categorical variable 1 = Yes, always 2 = Mostly 3 = Sometimes 4 = Seldom 5 = No, never		NBHW survey

### Data analysis

All statistical analyses were performed in R statistical software [34]. The analysis was carried out in four steps. *First*, descriptive statistics were used to examine the characteristics of the respondents regarding age, sex, and the prevalence of dementia. As data were missing for those who did not answer the survey questions, descriptive statistics were used to examine whether the respondents represented the underlying target population. We assumed the missing response mechanism to be completely at random [35], and the survey participant group was treated as a random sample from the target population. *Second*, descriptive statistics were used to examine the distribution of the survey answers for the dependent and independent variables over the three-year period. *Third,* ordinal logistic regression, or proportional odds (PO), models [36] were used to analyse associations between the dependent and independent variables. The dependent variables were analysed using six separate models, and the same independent variables were used in all models. To identify changes over the three-year period, the responses from 2016 were treated as the baseline for comparison with those from 2017 and 2018. The PO models were fitted by using the “polr” function from the MASS library [36] in R. All cases with missing data were excluded from the analysis. The response alternative “I do not know/no opinion” was treated as missing data. Approximately 72% of the respondents (*n* = 21 042) answered the survey for only one year between 2016 and 2018, 20% answered twice (*n* = 5 963), and 8% (*n* = 2 222) answered for all 3 years. Because the prevalence of repeated responses was low, the issue of intraclass correlation, due to repeated measures, was ignored in further analyses. *Fourth*, sensitivity analyses were performed using the group of respondents who had answered the survey for all three years (*n* = 2 222). Ordinal logistic regression, PO models, as described above, were used to analyse associations between the dependent and independent variables. PO models were fitted by Generalized estimating equation methods [37] using the “multgee” function from the multgee package [38] to adjust the inference for intraclass correlation due to repeated measures on the same individuals over three years.

## Results

### Description of the sample

A total of 13 763 (2016), 13 251 (2017) and 12 620 (2018) residents answered the survey. The majority of the respondents, 69%, were women, and the median age was 88 years. Approximately 80% of the respondents rated their health as good but had difficulties moving around by themselves. Of the respondents, approximately 20% were persons with dementia. Over the three years, the vast majority (94%) of the survey responses were received in paper format. The difference between the two groups of respondents (paper vs. online) with respect to their background (e.g., female, proportion with age 80 years or more, poor health condition) was very marginal, difference < 5% within any specific year. Throughout this article, these two response groups are assumed to be identical, which may not be an unreasonable assumption in the light of the descriptive statistics. Descriptive statistics for the independent and dependent variables are presented in Table 4.

**Table 4:** Descriptive statistics for the independent and dependent variables

	**2016** *n* = 13,763	**2017** *n* = 13 251	**2018** *n* = 12 620
**Independent variables**			
***Individual factors***			
*Age*			
Median	88	88	88
Q1-Q3	83–93	83–93	83–93
*Sex*			
1.Male	31%	32%	31%
2.Female	69%	68%	69%
*Dementia*			
1.No dementia	83%	80%	80%
2.Dementia	17%	20%	20%
*Self-rated health*			
1.Good	79%	78%	78%
2.Poor	21%	22%	22%
*Self-rated mobility*			
1.Can move around by myself	20%	20%	20%
2.Difficulties/cannot move around by myself	80%	80%	80%
***Attitudes of staff***			
Have you experienced any negative incidents in your contact with staff?			
1. Not experienced	1. 76%	1. 76%	1. 74%
2. Experienced	2. 24%	2. 24%	2. 26%
***Care environment***			
Do you thrive in your apartment?	1. 76% 2. 20% 3. 4%	1. 76% 2. 21% 3. 3%	1. 75% 2. 21% 3. 4%
Do you thrive in the public indoor areas?	1. 65% 2. 29% 3. 6%	1. 63% 2. 31% 3. 6%	1. 63% 2. 31% 3. 6%
Do you thrive in the outdoor areas?	1. 68% 2. 26% 3. 6%	1. 68% 2. 26% 3. 6%	1. 68% 2. 26% 3. 6%
Do you experience the mealtimes as a pleasant time of the day?	1. 25% 2. 44% 3. 20% 4. 8% 5. 3%	1. 24% 2. 44% 3. 20% 4. 8% 5. 4%	1. 24% 2. 44% 3. 20% 4. 9% 5. 3%
**Dependent variables**			
***Dignity***			
Do staff inform you beforehand about changes in your care?	1. 20% 2. 31% 3. 20% 4. 15% 5. 14%	1. 19% 2. 31% 3. 20% 4. 15% 5. 14%	1. 18% 2. 31% 3. 20% 4. 16% 5. 15%
Can you influence what time to get care?	1. 29% 2. 40% 3. 16% 4. 9% 5. 6%	1. 28% 2. 40% 3. 16% 4. 9% 5. 7%	1. 28% 2. 40% 3. 16% 4. 9% 5. 7%
Do staff consider your opinions and wishes regarding your care?	1. 38% 2. 43% 3. 13% 4. 4% 5. 2%	1. 38% 2. 43% 3. 13% 4. 4% 5. 2%	1. 37% 2. 43% 3. 14% 4. 4% 5. 2%
***Well-being***			
How safe or unsafe does it feel to live in the RCF?	1. 53% 2. 36% 3. 7% 4. 3% 5. 1%	1. 52% 2. 36% 3. 8% 4. 3% 5. 1%	1. 51% 2. 37% 3. 8% 4. 3% 5. 1%
Do you feel trust in staff at the RCF?	1. 45% 2. 42% 3. 12% 4. 1%	1. 44% 2. 42% 3. 13% 4. 1%	1. 42% 2. 43% 3. 14% 4. 1%
How satisfied or dissatisfied are you with the social activities offered at the RCF?	1. 27% 2. 40% 3. 22% 4. 7% 5. 4%	1. 27% 2. 41% 3. 22% 4. 6% 5. 4%	1. 28% 2. 40% 3. 21% 4. 7% 5. 4%

The response alternatives are presented in Table 2 and 3

### Factors associated with aspects of dignity and well-being over time (Models 1–6)

The associations between the dependent and independent variables over time are presented in Table 5 in terms of cumulative odds ratios (CORs) and 95% confidence intervals (CIs) from the six PO models.

**Table 5:** Cumulative odds ratios (CORs) and confidence intervals (CIs) from the six models

	**Model 1 Dignity**	**Model 2 Dignity**	**Model 3 Dignity**	**Model 4 Well-being**	**Model 5 Well-being**	**Model 6 Well-being**
	Do staff inform you beforehand about changes in your care?	Can you influence what time to get care?	Do staff consider your opinions and wishes regarding your care?	How safe or unsafe does it feel to live in the RCF?	Do you feel trust in staff at the RCF?	How satisfied or dissatisfied are you with the social activities offered at the RCF?
	COR (CI)	COR (CI)	COR (CI)	COR (CI)	COR (CI)	COR (CI)
**Independent variables**						
***Year***						
2016	Ref	Ref	Ref	Ref	Ref	Ref
2017	1.00 (0.95,1,06)	1.01 (0.95,1.06)	0.98 (0.93,1.04)	1.03 (0.97,1.09)	1.07 (1.01,1.13)^*^	0.94 (0.89,1.00)^*^
2018	1.05 (0.99,1.11)	0.99 (0.94,1.04)	1.01 (0.96,1.07)	1.01 (0.95,1.08)	1.13 (1.06,1.20)^*^	0.90 (0.85,0.95)^*^
***Individual factors***						
Age: 65–79 years	Ref	Ref	Ref	Ref	Ref	Ref
Age: 80 years	1.16 (1.09,1.23)^*^	1.09 (1.03,1.16)^*^	0.99 (0.93,1.05)	0.94 (0.88,1.01)	0.85 (0.79,0.91)^*^	0.93 (0.87,0.99)^*^
Sex: Male	Ref	Ref	Ref	Ref	Ref	Ref
Sex: Female	1.03 (0.98,1.08)	1.09 (1.04,1.47)^*^	0.90 (0.86,0.95)^*^	1.00 (0.94,1.05)	1.11 (1.05,1.17)^*^	0.85 (0.81,0.89)^*^
No dementia	Ref	Ref	Ref	Ref	Ref	Ref
Dementia	1.17 (1.10,1.24)^*^	1.52 (1.44,1.61)^*^	1.34 (1.26,1.42)^*^	1.14 (1.07,1.22)^*^	1.06 (0.99,1.13)	1.32 (1.24,1.39)^*^
Self-rated health: Good	Ref	Ref	Ref	Ref	Ref	Ref
Self-rated health: Poor	1.30 (1.22,1.37)^*^	1.46 (1.37,1.54)^*^	1.38 (1.30,1.47)^*^	1.51 (1.42,1.61)^*^	1.43 (1.35,1.53)^*^	1.34 (1.26,1.43)^*^
Self-rated mobility: Can move around by myself	Ref	Ref	Ref	Ref	Ref	Ref
Self-rated mobility: Difficulties/cannot move around by myself	1,22 (1.15,1.29)^*^	1.55 (1.47,1.65)^*^	1.32 (1.24,1.40)^*^	1.17 (1.09,1.25)^*^	1.18 (1.11,1.26)^*^	1.21 (1.14,1.28)^*^
***Attitudes of staff***						
Have you experienced any negative incidents in your contact with staff? Not experienced	Ref	Ref	Ref	Ref	Ref	Ref
Experienced	2.41 (2.28,2.55)^*^	2.76 (2.60,2.92)^*^	4.69 (4.41,4.99)^*^	3.22 (3.03,3.42)^*^	5.06 (4.74,5.39)^*^	1.70 (1.60,1.80)^*^
***Care environment***						
Do you thrive in your apartment?						
Yes	Ref	Ref	Ref	Ref	Ref	Ref
Partly	1.21 (1.14,1.28)^*^	1.58 (1.49,1.68)^*^	1.73 (1.62,1.84)^*^	2.88 (2.70,3.07)^*^	1.81 (1.70,1.94)^*^	1.49 (1.40,1.59)^*^
No	1.49 (1.29,1.72)^*^	2.15 (1.87,2.48)^*^	2.72 (2.35,3.14)^*^	5.96 (5.14,6.93)^*^	3.11 (2.65,3.64)^*^	2.23 (1.93,2.59)^*^
Are the public indoor areas pleasant?						
Yes	Ref	Ref	Ref	Ref	Ref	Ref
Partly	1.53 (1.45,1.62)^*^	1.26 (1.19,1.33)^*^	1.45 (1.37,1.54)^*^	1.84 (1.73,1.96)^*^	1.81 (1.70,1.92)^*^	2.02 (1.91,2.15)^*^
No	2.57 (2.29,2.88)^*^	1.78 (1.59,1.99)^*^	1.96 (1.74,2.20)^*^	2.83 (2.52,3.18)^*^	2.96 (2.62,3.34)^*^	4.62 (4.10,5.20)^*^
Are the outside areas pleasant?						
Yes	Ref	Ref	Ref	Ref	Ref	Ref
Partly	1.37 (1.30,1.44)^*^	1.49 (1.41,1.57)^*^	1.52 (1.44,1.61)^*^	1.48 (1.40,1.57)^*^	1.33 (1.25,1.40)^*^	1.74 (1.65,1.84)^*^
No	2.07 (1.87,2.29)^*^	1.87 (1.69,2.07)^*^	1.85 (1.66,2.05)^*^	1.75 (1.58,1.94)^*^	1.56 (1.40,1.74)^*^	2.85 (2.57,3.17)^*^
Do you experience the mealtimes as a pleasant time of the day?						
Yes, always	Ref	Ref	Ref	Ref	Ref	Ref
Mostly	2.15 (2.03,2.28)^*^	2.09 (1.97,2.21)^*^	2.37 (2.23,2.52)^*^	2.44 (2.27,2.62)^*^	2.74 (2.57,2.93)^*^	2.55 (2.40,2.70)^*^
Sometimes	3.57 (3.31,3.85)^*^	2.86 (2.65,3.09)^*^	3.53 (3.25,3.82)^*^	3.72 (3.41,4.06)^*^	4.49 (4.13,4.88)^*^	4.08 (3.77,4.42)^*^
Seldom	4.72 (4.25,5.24)^*^	3.16 (2.84,3.50)^*^	4.15 (3.71,4.63)^*^	5.11 (4.55,5.73)^*^	5.58 (4.97,6.26)^*^	5.51 (4.94,6.16)^*^
No, never	6.52 (5.54,7.66)^*^	3.67 (3.13,4.31)^*^	5.61 (4.74,6.64)^*^	5.76 (4.87,6.81)^*^	6.18 (5.20,7.34)^*^	7.01 (5.92,8.29)^*^

Inverse scale, COR > 1 indicate higher odds towards lower response category for a unit change in the respective independent variable

^*^ Significant at a level of 5%

For the factor of *time*, the COR for satisfaction with the aspects of dignity: information about changes in care, how to influence what time to receive care and how staff considered opinions and wishes regarding care, did not differ significantly over the three years. Regarding the aspects of well-being, the COR for satisfaction regarding how safe or unsafe it felt to live in the RCF, did not differ significantly over the three years. Regarding feeling trust in staff at the RCFs, there were significant differences over the years. The respondents in 2016 had higher COR of feeling trust in staff than the respondents in 2017 and 2018. This indicates that trust in staff decreased over the years. For social activities, there were differences over the years. The respondents in 2016 had lower COR of being satisfied with social activities than the respondents in 2017 and 2018.

The individual factors of *self-rated health, self-rated mobility* and *dementia* were significantly associated with the aspects of dignity and well-being over the three years. Respondents who rated their health as good, perceived no difficulties moving around by themselves and respondents without dementia had higher COR of being satisfied with the aspects of dignity and well-being.

For the individual factor *age*, there were associations over the years, as respondents aged 65–79 years had higher COR of being satisfied regarding aspects of dignity: information about changes in care and the possibilities to influence what time to receive care. In addition, a*ge* was associated with aspects of well-being, in that respondents aged 65–79 years had lower COR of feeling trust in staff at the RCF and also lower COR of being satisfied with social activities. For the individual factor *sex,* there were associations over the years, as men had higher COR of being satisfied regarding aspects of dignity: to influence what time to receive care, but lower COR of being satisfied with how staff considered their opinions and wishes. In addition, *sex* was associated with aspects of well-being over the years in that men had higher COR of feeling trust in staff at the RCF, but lower COR of being satisfied with social activities over the years.

*The attitudes of staff* were significantly associated with the aspects of dignity and well-being over the three years. Respondents who had not experienced disrespectful attitudes of staff had higher COR of being satisfied with the aspects of dignity and well-being.

The indoor *environment* was significantly associated with the aspects of dignity and well-being over the three years. Respondents who thrived in their apartments had higher COR of being satisfied with the aspects of dignity and well-being. The same tendencies were found for the public- and outdoor areas and the mealtime environment.

The sensitivity analyses for the group of respondents who had answered the survey for all three years showed the same tendencies for associations over the three years as described above. However, there were two differences. For *time,* there was one significant difference when comparing surveys from 2018 with those from 2016 regarding perceptions of how to influence what time to receive care. This significance was not found in the mixed cohort. Furthermore, for *time,* there were no significant differences regarding satisfaction with social activities when comparing surveys from 2018 and 2017 with those from 2016. This was significant in the mixed cohort. However, for these two differences, the estimated COR did not change by a large margin.

## Discussion

Our results identified associations between the dependent and independent variables over a three-year period. Residents who rated their health and mobility as good, residents without dementia, residents who had not experienced disrespectful attitudes of staff and residents who found the indoor-outdoor-mealtime environments to be pleasant had higher odds of being satisfied with aspects of dignity and well-being than their counterparts over the three-year period. These results strengthen previous cross-sectional research regarding factors associated with residents’ perceptions of aspects of dignity and well-being [27]. In addition, these results are consistent with previous research regarding the importance of evaluating factors influencing dignity among older persons living in RCFs [39]. Thus, the results can make an important contribution in identifying what factors should be targeted when designing sustainable improvement strategies to promote dignity and well-being in RCFs.

Our results identified that residents who rated their health as good, compared to residents who rated their health as poor, had higher odds of being satisfied with aspects of dignity and well-being over the years. In addition, residents without dementia had higher odds of being satisfied with aspects of dignity and well-being than residents with dementia. Regarding dementia, approximately 28% of persons with dementia in Sweden are living in RCFs [40]. Furthermore, approximately 67% of older persons living in RCFs have cognitive impairments [41]. Thus, health and dementia are important factors to target in improvement strategies to promote dignity and well-being. Regarding health, the staff in RCFs rate their competence in promoting health and well-being as low [42], and most managers in RCFs have a higher education in social care and not in health care [43]. This implies that managers may lack competence to support their staff in promoting residents’ health and well-being. Thus, RNs with a postgraduate degree in geriatric nursing could be used as facilitators in improvement strategies to promote health and well-being. However, only approximately 6% of RNs working in RCFs have a postgraduate degree in geriatric nursing in Sweden [44]. To promote health and well-being, there is a need to increase the number of RNs with this education. This is supported by the PCP framework describing the importance of the number of skilled staff with the requisite knowledge and skills to reach the person-centred outcomes of dignity and well-being [25, 26].

Our results identify that perceptions of aspects of dignity and well-being were associated with the attitudes of staff. These results are supported by the PCP framework describing the attitudes of staff as a prerequisite for reaching the person-centred outcomes of dignity and well-being [25, 26]. Thus, improvement strategies to promote dignity and well-being should focus on the attitudes of staff. The implementation strategy of the SNFVs targeted the attitudes of staff in the educational material provided by the NBHW [19, 20]. However, our results show that residents’ perceptions of aspects of dignity did not differ over the years and that the perceptions of trust in staff decreased over the three years. Why did this occur, even though an implementation strategy including education regarding attitudes was used? Education by itself is not sufficient as an implementation strategy, as changes in behaviour also must be considered [45, 46]. Therefore, a central issue in an implementation strategy is the question regarding how changes in behaviour can be facilitated. One part of the implementation strategy by the NBHW was that managers in RCFs had the opportunity to participate in a university course regarding how to support and facilitate the implementation of the SNFVs [20]. According to the integrated-Promoting Action on Research Implementation in Health Services (i-PARISH) framework, formal leadership support is central for implementation [47]. Leadership has also been described as important in facilitating the implementation of PCC [48–50]. However, the working situation for managers in RCFs is characterized by large groups of staff, making it problematic to influence norms and cultures [51]. Due to their strained work situations, it might be difficult for managers to prioritize facilitating behavioural change regarding the attitudes of staff.

Two of the core competencies of RNs are PCC and quality improvement work [52]. This implies that RNs have a responsibility to put the competencies into practice. Furthermore, RNs are covered by an ethical code stating their responsibility to promote an ethical approach by supporting and guiding staff to develop their ethical awareness [53]. Due to the responsibility of RNs in this area, we suggest that RNs could be used as facilitators in improvement strategies targeting the attitudes of staff. Our results also identify that the indoor-outdoor-mealtime environments were associated with satisfaction with aspects of dignity and well-being over the years. These results are supported by the PCP framework describing the care environment i.e., the physical environment as an important factor to consider for achieving the person-centred outcomes of dignity and well-being [25, 26]. As the environment is one of the meta-paradigms of nursing [54], we suggest that RNs could play an important role in improvement strategies targeting the environment at RCFs. However, there is a fairly low number of RNs working in RCFs [55], and many RNs experience not having enough time to fully perform care tasks due to a lack of resources [56]. RNs might therefore have a hard time prioritizing facilitating improvement work.

In sum, managers in RCFs have a responsibility to facilitate staff to put the SNFVs of dignity and well-being into practice [16, 43]. RNs have a higher education in nursing; the meta-paradigms of nursing are the human, health, environment and care [54]. Furthermore, two of the core competencies of RNs are PCC and quality improvement work [52]. We therefore suggest that these two professions collaborate to promote dignity and well-being in RCFs. The importance of collaboration between professionals has also been described in previous research as essential for implementing PCC [57]. However, we would like to highlight the importance of residents themselves being part of the collaboration when designing improvement strategies to promote dignity and well-being.

### Methodological limitations

A strength of this study was the use of national data. However, only 18–19% of the residents responded to the survey by themselves. The response rate is a limitation of this study [58]. There is also a limitation in that we do not know anything about the respondents who did not answer the survey. However, the descriptive statistics did not indicate any difference from the target population described in previous research [33, 40, 44]. One strength of this study was the use of longitudinal data; however, only 28% of the self-respondents answered the survey two or three times. However, this is a well-known problem when using longitudinal data regarding residents, as the average time for an older person to live in an RCF is approximately two years [44]. In this study, 80% of the respondents rated their health as good. It is possible that residents with poor self-rated health are underrepresented in this study. It should also be noted that approximately 20% of the respondents were diagnosed with/had prescribed medication for dementia. As previous research reports that approximately 67% of older persons living in RCFs have cognitive impairments [41], residents with dementia are also very likely to be underrepresented in this study. This possible underrepresentation of persons rating their health as poor and persons with dementia might impact the generalizability of the results. The generalizability might also be affected by the fact that we do not have any information about ethnicity/nationality of the respondents. Regarding the survey used for data collection, it was developed to support quality improvements in RCFs, but it has also been used in previous research [28]. For survey validity, it is critical to ensure that the questions in the survey measure what they are designed to measure [58]. When developing the survey, the NBHW used reference groups, cognitive interviews and a peer-review procedure [23]. However, a limitation in this study was that a scientific investigation on the reliability of the survey was missing. It should also be noted that due to the nonexperimental design of using observational data, the estimated effects may not be interpreted as causal effects. Despite these limitations, the results from this study can be valuable for improving care regarding promoting dignity and well-being in RCFs.

## Conclusions

The odds for satisfaction with aspects of dignity did not differ over the three-year period, but the odds for satisfaction with aspects of well-being, related to feeling trust in staff at the RCF, decreased over the years. Perceptions of aspects of dignity and well-being were associated with the attitudes of staff, the indoor-outdoor-mealtime environments and the individual factors of health, mobility, and dementia over time. Improvement strategies aiming to promote dignity and well-being need to target the associated factors. RNs, due to two of their core competencies, PCC and quality improvement work, should be given an active role as facilitators in improvement strategies to promote dignity and well-being in RCFs. The results from this study can be used by RNs and managers in RCFs when designing improvement strategies. In addition, as our results regarding the associated factors confirm the PCP framework, we suggest that it could be used as a theoretical framework when designing improvement strategies to promote dignity and well-being.

## Data Availability

The datasets used and analyzed during the current study are available from the corresponding author on reasonable request.

## Abbreviations

CI: Confidence interval
COR: Cumulative odds ratio
i-PARISH: Integrated-Promoting Action on Research Implementation in Health Services
NA: Nurse assistant
NBHW: National Board of Health and Welfare
PCC: Person-centred care
PCP framework: Person-centred practice framework
PO: Proportional odds
Q: Quartile
RCF: Residential care facility
RN: Registered nurse
SNFV: The Swedish national fundamental values
